# Voltage‐gated calcium channels and their auxiliary subunits: physiology and pathophysiology and pharmacology

**DOI:** 10.1113/JP272262

**Published:** 2016-07-05

**Authors:** Annette C. Dolphin

**Affiliations:** ^1^ Department of Neuroscience, Physiology and PharmacologyUniversity College LondonGower StreetLondonWC1E 6BTUK

## Abstract

Voltage‐gated calcium channels are essential players in many physiological processes in excitable cells. There are three main subdivisions of calcium channel, defined by the pore‐forming α_1_ subunit, the Ca_V_1, Ca_V_2 and Ca_V_3 channels. For all the subtypes of voltage‐gated calcium channel, their gating properties are key for the precise control of neurotransmitter release, muscle contraction and cell excitability, among many other processes. For the Ca_V_1 and Ca_V_2 channels, their ability to reach their required destinations in the cell membrane, their activation and the fine tuning of their biophysical properties are all dramatically influenced by the auxiliary subunits that associate with them. Furthermore, there are many diseases, both genetic and acquired, involving voltage‐gated calcium channels. This review will provide a general introduction and then concentrate particularly on the role of auxiliary α_2_δ subunits in both physiological and pathological processes involving calcium channels, and as a therapeutic target.

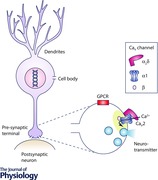

**Abbreviations**
AID: α‐interaction domain
AP‐1: adaptor protein complex‐1
BBS: bungarotoxin binding site
BTX: α‐bungarotoxin
DRG: dorsal root ganglion
EM: electron microscopy
ER: endoplasmic reticulum
GK: guanylate kinase
GPCR: G‐protein coupled receptor
GPI: glycosyl‐phosphatidyl inositol
HIV: human immunodeficiency virus
MIDAS: metal ion‐dependent adhesion site
PMCA: plasma membrane Ca^2+^‐ATPase
RyR: ryanodine receptor
SERCA: sarcoplasmic and endoplasmic reticulum Ca^2+^ ATPase
SH3: src homology‐3
SNP: single nucleotide polymorphism
VWA: Von Willebrand Factor‐A domain

## Introduction

Excitable cells contain functional voltage‐gated ion channels, including calcium channels. Neurons and muscle cells are conventionally excitable, but many other cell types show oscillatory changes in voltage, dependent on the interplay between voltage‐gated and calcium‐dependent channels (for example see Hu *et al*. 2012). Free intracellular Ca^2+^ is maintained at 10–100 nm in the cytoplasm, a low level relative to the extracellular milieu. Voltage‐gated calcium channels then react to membrane depolarization by opening, and thus allowing Ca^2+^ to move down its electrochemical gradient. Ca^2+^ entry, particularly but not exclusively through voltage‐gated calcium channels, provides an elevation of intracellular calcium ion concentration, to drive many processes. These include hormone secretion, neurotransmitter release, calcium‐dependent transcription of a variety of genes, and also spontaneous pacemaker activity in some neurons, muscles and secretory cells (Mangoni *et al*. 2003; Guzman *et al*. 2009; Putzier *et al*. 2009; Hu *et al*. 2012; Striessnig *et al*. 2015). The present review concentrates particularly on the roles of the accessory α_2_δ subunits. For more comprehensive coverage of calcium channel function, the reader is directed to other recent reviews (Striessnig *et al*. 2014; Zamponi *et al*. 2015; Zamponi, 2016).

## Voltage‐gated calcium channel subunits

**Figure 1:**
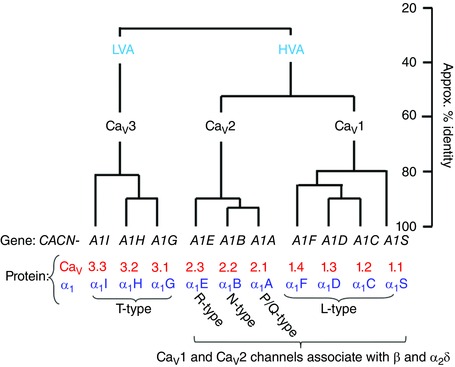
**Calcium channel α_1_ subunit homology** The relationship between the 10 mammalian voltage‐gated calcium channel α_1_ subunits, and their gene names (black) and protein nomenclature (red and blue). The calcium channels were historically first divided into high voltage‐activated (HVA) and low voltage‐activated (LVA).

Functional voltage‐gated calcium channels are composed of pore‐forming α_1_ subunit proteins, encoded by the *CACNA1x* genes (for review see Catterall *et al*. 2003), of which there are 10 isoforms in the mammalian genome. In the case of the Ca_V_1.1–Ca_V_1.4 channels (known as L‐type channels), these are encoded by *CACNA1S, ‐C, ‐D* and *‐F*, respectively, and also known as α_1_S, α_1_C, α_1_D and α_1_F. The Ca_V_2.1–Ca_V_2.3 channels (termed P/Q ‐, N‐and R‐type from physiological experiments: Nowycky *et al*. 1985; Mintz *et al*. 1992; Piedras‐Rentería & Tsien, 1998) are encoded by *CACNA1A, ‐B* and *‐E*, respectively, and also known as α_1_A, α_1_B and α_1_E. The T‐type Ca_V_3 channels (encoded by *CACNA1G, ‐H* and ‐*I*) are also termed α_1_G, α_1_H and α_1_I (Cribbs *et al*. 1998; Perez‐Reyes *et al*. 1998). They are much more similar to each other than to the Ca_V_1 and Ca_V_2 channels (Fig. 1).

**Figure 2:**
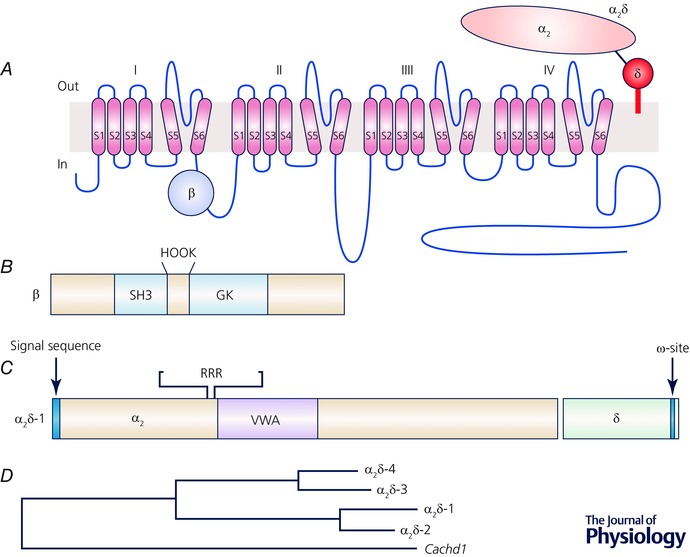
**Domains in β and α_2_δ subunits and their interaction with the α_1_ subunit** *A*, topology of the calcium channel complex. *B*, known domains in β subunits. *C*, known domains in α_2_δ subunits. *D*, approximate phylogenetic tree generated for mouse α_2_δ subunits using http://www.phylogeny.fr/. The VWA and Cache containing protein Cachd1 is included for comparison. The sequences AAI15872.1, AAH56389.1, EDL24740.1, AAI41092.1 and NP_932154.1 were used.

Although the α_1_ subunits dictate the principal biophysical and pharmacological properties of these channels, their expression is enhanced and their properties are modified by the two main auxiliary (or accessory) subunits (Tanabe *et al*. 1987; Mikami *et al*. 1989; Mori *et al*. 1991; Varadi *et al*. 1991). The α_2_δ and β subunits also play important roles in channel folding and their subsequent transport to the cell surface, and into particular domains of polarized cells such as neurons. These processes are together known as trafficking, and involve multiple steps. Both the Ca_V_1 and Ca_V_2 classes of channels are able to form a heteromeric complex, co‐assembling with one of four β subunits (encoded by *CACNB1—4*; Fig. 2 *A* and *B*), and one of four α_2_δ subunits (encoded by *CACNA2D1—4;* Fig. 2 *A* and *C*). For the Ca_V_3 channels, the α_1_ subunits can form functional channels alone, but may also associate with other proteins.

All of the α_1_, β and α_2_δ subunits form a large number of variants as a consequence of alternative splicing events. This opens the potential for a huge diversity of properties and function. A γ subunit also forms part of the skeletal muscle calcium channel complex, which comprises Ca_V_1.1, β1a, γ1 and α_2_δ‐1(Jay *et al*. 1990). However, although multiple other γ subunits have been cloned (Letts *et al*. 1998; Moss *et al*. 2002; Tomita *et al*. 2003), no γ subunits have been shown to form an integral part of cardiac (Walsh *et al*. 2009) or neuronal (Moss *et al*. 2002; Müller *et al*. 2010) calcium channels. In contrast, they have well‐defined roles as transmembrane AMPA‐glutamate receptor modifying proteins (Tomita *et al*. 2003). Furthermore, for some Ca_V_1 and Ca_V_2 calcium channels, the tight binding of calmodulin to the so‐called ‘IQ’ domain in their C‐terminal tail allows calmodulin to be considered as a quasi‐subunit (Mori *et al*. 2008; Kim *et al*. 2010; Ben‐Johny *et al*. 2013).

## Voltage‐gated calcium channel localization

Ca_V_1.1 is the only isoform present in mammalian skeletal muscle t‐tubules, and shows very low expression elsewhere, including brain (Bannister & Beam, 2013). Ca_V_1.2 is the main isoform in ventricular cardiac muscle, and is also present in smooth muscle cells, secretory tissue and the nervous system (Striessnig *et al*. 2014). Ca_V_1.3 has a more limited localization than Ca_V_1.2, playing a major role in sinoatrial node tissue, and in the auditory system (Platzer *et al*. 2000; Mangoni *et al*. 2003; Baig *et al*. 2011), although it is also present in brain. It is also present in some endocrine tissues, including aldosterone‐secreting cells of the adrenal gland, where somatic mutations give rise to resistant hypertension (Azizan *et al*. 2013; Scholl *et al*. 2013). Ca_V_1.4 shows very restricted distribution, particularly in the visual system (Mansergh *et al*. 2005).

The Ca_V_2.*x* channels show a primarily neuronal distribution and are involved in fast neurotransmitter release (Takahashi & Momiyama, 1993; Wu *et al*. 1999; Cao & Tsien, 2010). Ca_V_2.1 channels are present throughout the brain, and are particularly prevalent in cerebellum (Ophoff *et al*. 1996), where they make up the predominant calcium current in Purkinje neurons (Mintz *et al*. 1992; Westenbroek *et al*. 1995). They are involved in neurotransmission in most mature mammalian central synapses (Westenbroek *et al*. 1995; Iwasaki *et al*. 2000, 2005; Nakamura *et al*. 2015). Ca_V_2.2 is distributed throughout the central (Westenbroek *et al*. 1992) and peripheral nervous systems (Lipscombe *et al*. 1988; Boland *et al*. 1994; Wheeler *et al*. 1994). It is particularly important for neurotransmission early in mammalian development, although it co‐exists with Ca_V_2.1 in most mature synapses (Iwasaki *et al*. 2000). Ca_V_2.2 also plays a dominant role in the mature peripheral nervous system (Chaplan *et al*. 1994; Bowersox *et al*. 1996). Ca_V_2.3, although originally described as being low voltage activated (Soong *et al*. 1993), is thought to correspond to residual R‐type calcium current (Zhang *et al*. 1993; Tottene *et al*. 2000; Wilson *et al*. 2000). It is present in many brain regions and is found both pre‐ and postsynaptically in neurons (Parajuli *et al*. 2012). Ca_V_2.3 has been found to be involved in spontaneous release of glutamate (Ermolyuk *et al*. 2013), although the Ca_V_2.3 blocker SNX‐482 also blocks some K^+^ channels, making dissection of its physiological functions more difficult (Kimm & Bean, 2014).

The Ca_V_3 channels are extensively distributed in neurons and other excitable cells (Cribbs *et al*. 1998; Perez‐Reyes, 1998; Perez‐Reyes *et al*. 2009). For example, they are prevalent in the thalamus (Perez‐Reyes, 2003), and also have important roles in primary afferent pathways (Francois *et al*. 2015; Gadotti *et al*. 2015; for recent review see Zamponi *et al*. 2015). They have important roles in neuronal and cardiac excitability and in cardiac and neuronal pacemaker activity (Perez‐Reyes, 2003; Guzman *et al*. 2009; Putzier *et al*. 2009). In some synapses they also have a presynaptic function in transmitter release (Huang *et al*. 2011; Carbone *et al*. 2014).

## Association of α_1_ subunits with auxiliary subunits

Biochemical isolation of calcium channels has indicated that native L‐, N ‐ and P/Q ‐type channels in muscle and brain are all associated with β and α_2_δ subunits (Tanabe *et al*. 1987; Witcher *et al*. 1993; Liu *et al*. 1996). However, it has been noted that the association of the α_2_δ subunit with the channel complex is more easily dissociated by the detergents used during purification than the interaction of the β subunit (Jay *et al*. 1991; Gee *et al*. 1996; Müller *et al*. 2010). It is also possible that not all native calcium channel complexes contain an α_2_δ subunit. By contrast the association between the α_1_ and β subunits is quite robust, and shows a high affinity for interaction with the intracellular loop between domains I and II of Ca_V_1 and Ca_V_2 channels (Pragnell *et al*. 1994; Canti *et al*. 2001; Van Petegem *et al*. 2004). Despite this difference, both the β and α_2_δ subunits increase the expression and function of these channels, as described below.

## Structural information on voltage‐gated calcium channels

There is detailed structural information concerning the cytoplasmic β subunits. Initially a modelling study showed that β subunits contained a core SH3 and guanylate kinase‐like (GK) domain (Hanlon *et al*. 1999; Fig. 2 *B*). This was confirmed in X‐ray crystallographic studies of the SH3‐GK core domains of three calcium channel β subunits, in association with an interacting peptide derived from the I‐II linker (Chen *et al*. 2004; Opatowsky *et al*. 2004; Van Petegem *et al*. 2004). From these and other studies, the GK domain is seen to bind to the α‐interaction domain (AID) which is in the proximal part of the I‐II linker (Fig. 2 *A*). The β subunit is thought to promote the formation of an α‐helix, in the AID motif, extending back to the end of S6 in domain I (Opatowsky *et al*. 2004; for reviews see Richards *et al*. 2004; Buraei & Yang, 2010). This is likely to promote folding to form mature channels.

More recently, very valuable crystallographic information pertaining to the α_1_ subunit structure has come from studies of the bacterial single domain sodium channel Na_V_Ab, whose structure was solved by X‐ray crystallography (Payandeh *et al*. 2011). Subsequently, key residues in the pore of this channel were mutated to render the channel Ca^2+^ permeable (Tang *et al*. 2014); this structure was able to provide detailed information about the Ca^2+^ permeation pathway. There are also structures of calmodulin interacting with the proximal C‐terminus of Ca_V_1.2 (Kim *et al*. 2008, 2010), revealing the nature of this interaction and shedding light on the mechanism of Ca^2+^‐dependent inactivation.

The initial low resolution single particle electron micrographic (EM) structures of the L‐type calcium channel complex, also called the dihydropyridine receptor, from skeletal muscle (Serysheva *et al*. 2002; Wolf *et al*. 2003; Wang *et al*. 2004; Hu *et al*. 2015) and cardiac muscle (Walsh *et al*. 2009), showed an asymmetric structure, with a density identified as α_2_δ extending out from the complex. More recently a high resolution cryo‐EM structure of the Ca_V_1.1 calcium channel complex purified from skeletal muscle has now provided us with much greater detail, at near atomic resolution, particularly regarding the transmembrane organization and pore of the α_1_ subunit, and the orientation of the α_2_δ subunit domains (Wu *et al*. 2015). It has shown a clockwise arrangement of the α_1_ subunit domains, and identified that there are multiple interactions of α_2_δ‐1 subunit with the extracellular loops of domains I‐III of the α_1_S subunit.

## Modulation of calcium channel function by second messengers and G proteins

There is insufficient space in this review to cover the enormous amount of information on multiple second messenger effects on calcium channel function. Three key areas that can be highlighted are firstly: Ca^2+^‐dependent inactivation and facilitation of Ca_V_2.1, Ca_V_1.2 and Ca_V_1.3 channels, by interaction with calmodulin associated with the C‐terminal tail of the α_1_ subunit (Dick *et al*. 2008; Minor & Findeisen, 2010; Ben‐Johny *et al*. 2013). Secondly, there is an important phosphorylation process that is responsible for β‐adrenergic stimulation of cardiac calcium currents (Reuter, 1987; Fuller *et al*. 2010). The mechanism involves enhancement of Ca_V_1.2 currents by cyclic AMP‐dependent protein kinase, which results from phosphorylation‐induced relief of auto‐inhibition by a peptide cleaved from the channel C‐terminus (Fuller *et al*. 2010; Fu *et al*. 2013). Thirdly, there is a ubiquitous G‐protein coupled receptor (GPCR)‐mediated inhibition of the Ca_V_2 class of channels mediated by Gβγ (Dolphin, 2003; Zamponi & Currie, 2013).

Regarding the interplay between second messenger modulation and auxiliary subunits, initial studies identified that Gβγ bound to a site on the I‐II linker of Ca_V_2 channels that overlapped with the Ca_V_β subunit (Zamponi *et al*. 1997), opening the possibility that they compete for this binding site. We then identified that in the absence of a Ca_V_β subunit, Gβγ‐mediated inhibition is still present, but it is not voltage dependent, meaning that it cannot be removed by preceding depolarization. Therefore, the presence of the Ca_V_β subunit is required for Gβγ‐mediated G‐protein modulation to show voltage‐dependent properties (Meir *et al*. 2000; Zhang *et al*. 2008), and a simple competition for binding is not responsible for Gβγ‐mediated inhibition. Further to this, we identified key residues within the N‐terminus of Ca_V_2 channels that are essential for G‐protein modulation (Page *et al*. 1998; Canti *et al*. 1999; Leroy *et al*. 2005), and this work was extended by others (Agler *et al*. 2005).

## Interplay between the action of β and α_2_δ subunits in calcium channel function

**Figure 3:**
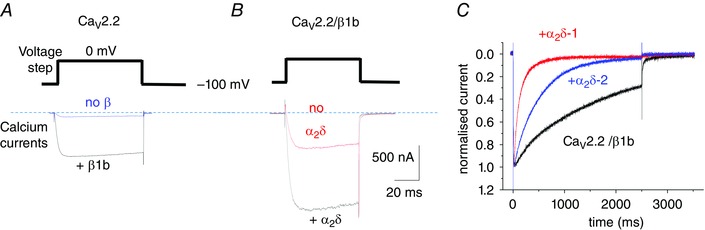
**Examples of effects of auxiliary subunits on Ca_V_2.2 calcium channel currents** *A*, Ca_V_2.2 calcium currents: effect of β subunits. Example of peak Ca_V_2.2 current at 0 mV in the absence of β (blue) and presence of β1b (black). *B*, Ca_V_2.2 calcium currents: effect of α_2_δ subunits. Example of peak Ca_V_2.2/β1b current at 0 mV in the absence of α_2_δ (red) and presence of α_2_δ‐3 (black). Scale bars apply to both *A* and *B*. Charge carrier 1 mm Ba^2+^, expression in tsA‐201 cells, as in a previous study (Leroy *et al*. 2005). *C*, effect of different α_2_δ subunits on inactivation. Examples of normalized peak current for Ca_V_2.2–β1b (black), Ca_V_2.2–β1b–α_2_δ‐2 (blue) and Ca_V_2.2–β1b–α_2_δ‐1 (red), over a 2.5 s timescale. Expression in *Xenopus* oocytes, as in a previous study (Canti *et al*. 2005).

**Figure 4:**
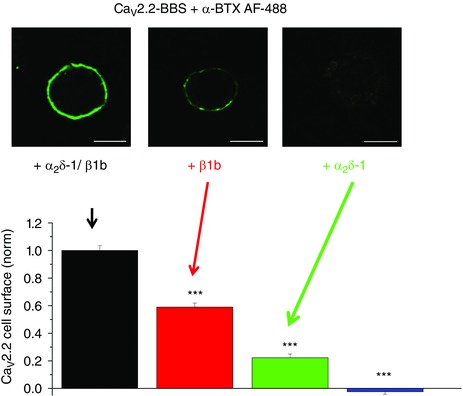
**Ca_V_2.2 cell surface expression: effects of β1b and α_2_δ‐1** Cell surface expression of bungarotoxin binding site (BBS) tagged Ca_V_2.2 labelled with α‐bungarotoxin (BTX) coupled to AF488 dye (green). Top panel: examples of N2a cells transfected with Ca_V_2.2–β1b–α_2_δ‐1 (left), Ca_V_2.2–β1b (middle) and Ca_V_2.2–α_2_δ‐1 (right). Scale bar 20 μm. Bottom panel: mean (± SEM) data for cell surface expression of Ca_V_2.2, for cells expressing Ca_V_2.2–β1b–α_2_δ‐1 (black bar), Ca_V_2.2–β1b (red bar) and Ca_V_2.2–α_2_δ‐1 (green bar) or Ca_V_2.2 alone (blue bar). Data are taken from a recent study (Cassidy *et al*. 2014).

For both Ca_V_1 and Ca_V_2 channels, the Ca_V_β subunits are extremely important for expression of functional channels in several heterologous expression systems (Varadi *et al*. 1991; Pragnell *et al*. 1994; Jones *et al*. 1998; Leroy *et al*. 2005). Interaction of the α_1_ subunit with a β subunit has a number of consequences. By binding via their guanylate kinase domain (Fig. 2 *B*) to the intracellular AID motif on the α_1_ subunits (Pragnell *et al*. 1994; Fig. 2 *A*), they increase folding of the channels and protect the channels from endoplasmic reticulum (ER)‐associated proteasomal degradation (Altier *et al*. 2011; Waithe *et al*. 2011); thus they allow more channels to reach the plasma membrane (Fig. 3 *A*). However, it is difficult to determine whether β subunits are absolutely essential for α_1_ subunits to reach the cell surface. This suffers from the problem that several expression systems, in particular *Xenopus* oocytes, express native β subunits (Canti *et al*. 2001). The α_2_δ subunits produce an additional increase in current density, described in more detail below (Fig. 3 *B*). However, because a number of expression systems, including *Xenopus* oocytes, HEK‐293 and the tsA‐201 cells derived from them, also contain some endogenous α_2_δ‐1 (Singer‐Lahat *et al*. 1992; Dolphin *et al*. 1999; Kadurin *et al*. 2012 *a*), this also complicates assessment of their role. Nevertheless, both α_2_δ and β subunits increase the expression at the plasma membrane of Ca_V_1 and Ca_V_2 channels, and where it has been investigated, some evidence suggests that α_2_δ subunits are poorly effective unless the Ca_V_β subunits are also expressed (Cassidy *et al*. 2014; Fig. 4).

## Isoforms and topology of α_2_δ

All the α_2_δ proteins have a similar structure (for reviews see Felix, 1999; Davies *et al*. 2007; Dolphin, 2012; Fig. 2 *C* and *D*). The N‐terminus has a signal sequence directing the nascent polypeptide into the lumen of the ER, such that it becomes extracellular, once transported to the plasma membrane (Fig. 2 *C*). Several domains can be identified in the sequence of α_2_δ proteins, including a Von Willebrand Factor‐A (VWA) domain (Whittaker & Hynes, 2002; Fig. 2 *C*). These domains, as well as being present in von Willebrand factor itself, are generally involved in extracellular protein–protein interactions, dependent on divalent cations, particularly by integrins and extracellular matrix proteins. A key motif in VWA domains is the metal ion‐dependent adhesion site (MIDAS), which involves coordination of the divalent cation by a ring of up to five polar or charged residues (Whittaker & Hynes, 2002). If the MIDAS site is ‘perfect’, with the full complement of five residues, it is highly likely to be involved in such protein–protein interactions (Whittaker & Hynes, 2002), and this is the case in α_2_δ‐1 and α_2_δ‐2 (Canti *et al*. 2005), whereas α_2_δ‐3 and α_2_δ‐4 have one missing polar residue in the MIDAS motif. There is also a region in the α_2_δ subunits containing so‐called Cache domains, which have homology to bacterial chemosensory domains (Anantharaman & Aravind, 2000; Dolphin, 2012). The recent EM structure also identified a Cache domain, N‐terminal to the VWA domain (Wu *et al*. 2015). There are other identified genes with predicted similarity to α_2_δ subunits, such as *CACHD1* (Whittaker & Hynes, 2002) (Fig. 2 *D*), whose functions remain to be determined.

The C‐termini of all α_2_δ subunits all have a hydrophobic region first identified to be a transmembrane domain (Ellis *et al*. 1988; Jay *et al*. 1991). This led to the α_2_δ proteins being described as single pass type I (C‐terminal cytoplasmic) transmembrane proteins. From prediction progams we found that at least two of the α_2_δ subunits (α_2_δ‐3 and α_2_δ‐4) are predicted with high likelihood to be glycosyl‐phosphatidyl inositol (GPI)‐anchored, partly because the C‐terminal hydrophobic domain is very short and present at the extreme C‐terminus, as well as the presence of a predicted GPI‐anchor ω‐site (Pierleoni *et al*. 2008; Davies *et al*. 2010). We have provided evidence for this post‐translational modification for α_2_δ‐1, α_2_δ‐2 and α_2_δ‐3 (Davies *et al*. 2010; Alvarez‐Laviada *et al*. 2014). The genes for all the α_2_δ subunits encode a single precursor protein, which is post‐translationally proteolytically processed into two polypeptides (Jay *et al*. 1991). The α_2_ and δ polypeptides remain disulfide‐bonded together. The cysteines residues involved in this disulfide bonding have been determined for α_2_δ‐1 (Calderon‐Rivera *et al*. 2012). We have recently been studying the relevance of proteolytic processing into α_2_ and δ to the physiological function of α_2_δ (Kadurin *et al*. 2012 *b*, and authors’ unpublished data).

## **Effect of** α_2_δ **subunits on calcium channels reaching the plasma membrane**

In general, the α_2_δ subunits have been found to increase the expression (either functional expression or amount of protein on the plasma membrane) of several different Ca_V_1 or Ca_V_2 combinations with β subunits (Shistik *et al*. 1995; Gurnett *et al*. 1996; Felix *et al*. 1997; Wakamori *et al*. 1999; Gao *et al*. 2000; Yasuda *et al*. 2004; Canti *et al*. 2005; Davies *et al*. 2010). For example, for the Ca_V_2.1–β4 combination, calcium currents were increased 3‐fold by α_2_δ‐2. This set of calcium channel subunits is found in cerebellar Purkinje cells, where α_2_δ‐2 is strongly represented (Barclay *et al*. 2001; Brodbeck *et al*. 2002). However, α_2_δ‐2 did not alter the single channel conductance, suggesting strongly that the large increase in whole cell current is solely due to an increase in the number of functional channels at the cell surface (Barclay *et al*. 2001; Brodbeck *et al*. 2002). However, the term ‘increased number of functional channels’ can indicate increased amount of channel protein in the plasma membrane and/or an increased proportion of the channels already in the plasma membrane able to respond to depolarization. There is strong evidence that the cell surface expression of Ca_V_1 and Ca_V_2 α_1_ subunits is increased by α_2_δ subunits (Fig. 4), although the mechanism(s) underlying this increase this are still being unravelled (Tran‐Van‐Minh & Dolphin, 2010; Cassidy *et al*. 2014).

The situation for Ca_V_2.3 channels is less clear. It has been reported that Ca_V_2.3 produces relatively large currents when expressed alone in *Xenopus* oocytes (Soong *et al*. 1993; Schneider *et al*. 1994; Qin *et al*. 1998), and α_2_δ‐1 subunits did not increase Ca_V_2.3 currents in this expression system (Qin *et al*. 1998). Furthermore, in HEK‐293 cells α_2_δ‐1 produced a 2‐fold elevation of the maximum conductance for Ca_V_2.3 alone, although it gave no additional increase beyond that of β subunits (Jones *et al*. 1998). Thus it is possible that Ca_V_2.3 may be less affected by α_2_δ subunits, but this will require confirmation.

Increased trafficking of the calcium channels by these α_2_δ subunits is highly likely not to be their only mechanism of action. For example, liposomes containing skeletal muscle calcium channel protein exhibited greater calcium flux in the presence of α_2_δ subunits than in their absence (Gutierrez *et al*. 1991). Furthermore, the effect of α_2_δ‐1 on Ca_V_2.2 channel density in the plasma membrane when expressed in N2a cells was at the most 2‐fold (Cassidy *et al*. 2014), whereas there was an approximately 10‐fold increase in Ca_V_2.2 calcium currents in the presence of α_2_δ‐1 (Hoppa *et al*. 2012). It has been suggested that α_2_δ‐1 reduced the apparent turnover of Ca_V_2.2, in studies using radiolabelled conotoxin (Bernstein & Jones, 2007), although Cassidy *et al*. (2014) did not find that α_2_δ‐1 reduced Ca_V_2.2 endocytosis from the plasma membrane in N2a cells.

The perfect MIDAS motif present in the VWA domain of α_2_δ‐1 and α_2_δ‐2 subunits is required for increasing calcium currents (Canti *et al*. 2005; Hoppa *et al*. 2012), and also for cell surface expression of Ca_V_2.2 (Cassidy *et al*. 2014). Mutation of the MIDAS motif also reduced the trafficking of the α_2_δ subunits themselves, when expressed alone (Canti *et al*. 2005; Cassidy *et al*. 2014). This mutation also abolished the capacity of both α_2_δ‐1 (Hoppa *et al*. 2012) and α_2_δ‐2 (Canti *et al*. 2005) subunits to increase calcium currents in several expression systems. However, α_2_δ‐3 and α_2_δ‐4 do not contain perfect MIDAS motifs (Whittaker & Hynes, 2002), and may therefore play a smaller trafficking role, despite increasing calcium currents (Davies *et al*. 2010), by what must be an additional mechanism.

In an early study, it was found that the α_2_ subunit of α_2_δ‐1 binds to of Ca_V_1.1 domain III (Gurnett *et al*. 1997). However, the recent structural study shows interaction of α_2_δ‐1 with several extracellular loops in domains I–III of Ca_V_1.1 (Wu *et al*. 2015). The α_2_δ‐1 MIDAS motif was found to be located immediately above the linker between the first two transmembrane segments in voltage‐sensing domain I (Wu *et al*. 2015). The limited structural evidence for other calcium channels also suggests extensive extracellular contact between Ca_V_1.2 and α_2_δ‐1 (Walsh *et al*. 2009).

The Ca_V_3 calcium channels produce large currents in the absence of co‐expressed accessory β or α_2_δ subunits, and therefore these proteins are not obligate auxiliary subunits for Ca_V_3 channels. Nevertheless, both α_2_δ‐1 and α_2_δ‐2 were found to increase Ca_V_3.1 currents and cell surface expression almost 2‐fold (Dolphin *et al*. 1999; Gao *et al*. 2000; Dubel *et al*. 2004); thus these channels may have the capacity to associate with α_2_δ subunits. In contrast, other studies found that α_2_δ‐1 and α_2_δ‐3 produced little change, whereas α_2_δ‐2 had a larger effect on Ca_V_3.1 current density (Klugbauer *et al*. 1999; Lacinova *et al*. 1999; Hobom *et al*. 2000).

## Trafficking of calcium channels to specific membrane domains

The auxiliary α_2_δ and β subunits play major roles in the trafficking of Ca_V_1 and Ca_V_2 channels not only to the cell surface, but also to specific domains of polarized cells, including muscle cells and neurons (Dolphin, 2012; D'Arco *et al*. 2015). We have postulated that the α_2_δ subunits are highly likely to interact with proteins involved in trafficking of membrane protein cargoes (Davies *et al*. 2006; Hendrich *et al*. 2008; Tran‐Van‐Minh & Dolphin, 2010). We have found that the α_2_δ subunits themselves purify with cholesterol‐rich lipid raft domains, and this may influence localization of the calcium channel complexes in plasma membrane microdomains (Davies *et al*. 2006, 2010; Kadurin *et al*. 2012 *a*). Interestingly, we have also found that a truncated α_2_δ subunit, from which we have removed the C‐terminal GPI‐anchor motif, is mainly secreted, but nevertheless exhibits some extrinsic plasma membrane association, via interactions that remain to be determined (Kadurin *et al*. 2012 *a*).

In recent work, we have found that the adaptor protein complex‐1 (AP‐1) is important for trafficking of Ca_V_2.2 from the trans‐Golgi network to the plasma membrane, via an alternatively spliced exon 37 in the proximal C‐terminus. The splice variant of Ca_V_2.2 containing exon 37a supports larger currents compared to that containing exon 37b (Castiglioni *et al*. 2006), and is selectively expressed in nociceptors (Bell *et al*. 2004). Our work revealed that AP‐1 binding motifs, YxxΦ and [DE]xxxL[LI], present only in exon 37a, increase the intracellular trafficking of exon 37a‐containing Ca_V_2.2, both to the somatic plasma membrane and into the axons of dorsal root ganglion (DRG) neurons. The ability of exon37a to increase Ca_V_2.2 currents and cell surface density are lost in the absence of α_2_δ subunits, suggesting that this auxiliary subunit promotes a particular step in the forward trafficking process (Macabuag & Dolphin, 2015).

## Influence of α_2_δ subunits on biophysical properties of calcium channels

The α_2_δ subunits influence the voltage‐dependent and kinetic properties of the calcium currents; in particular they consistently increase the inactivation rate, although to different extents. The effects of α_2_δ subunits may also depend on the presence of a particular β subunit.

### Activation

In the case of Ca_V_1.2, it was found that α_2_δ‐1 subunits exerted little effect on the activation voltage dependence (Singer *et al*. 1991; Welling *et al*. 1993; Shistik *et al*. 1995; Bangalore *et al*. 1996; Shirokov *et al*. 1998). However, in other studies a hyperpolarization of activation was reported (Felix *et al*. 1997), and this was also observed from conductance‐voltage measurements (Platano *et al*. 2000). For Ca_V_2.1, co‐expressed with β4 in mammalian cells, α_2_δ‐2 did not affect the voltage dependence of activation (Brodbeck *et al*. 2002). For Ca_V_2.2 co‐expressed with β1b, α_2_δ‐1 increased the activation rate of currents, but had less effect on the voltage dependence of activation (Wakamori *et al*. 1999). Contrasting results were found for Ca_V_2.3, which shows a greater capacity than Ca_V_1.2 to produce currents in the absence of the auxiliary subunits (Stephens *et al*. 1997; Qin *et al*. 1998). For Ca_V_2.3, α_2_δ‐1 was found to depolarize the activation, in the presence of either β1b or β2a, or in the absence of any β subunits (Qin *et al*. 1998). In contrast, in another study α_2_δ‐1 had no effect on the activation voltage dependence for Ca_V_2.3 (Jones *et al*. 1998).

### Inactivation

In some studies, it was found that the α_2_δ subunits hyperpolarized the steady‐state inactivation for several different calcium channel isoforms (Singer *et al*. 1991; Felix *et al*. 1997; Wakamori *et al*. 1999; Hobom *et al*. 2000; Canti *et al*. 2005; Hendrich *et al*. 2008; Davies *et al*. 2010), and in α_2_δ‐1 knockout mice there was a clear depolarization of the steady‐state inactivation curve for cardiac calcium channel currents (Fuller‐Bicer *et al*. 2009). However, for Ca_V_2.3 it was found that, whereas β1b caused a hyperpolarization of the steady‐state inactivation, α_2_δ‐1 had no effect on this, either with or without a β subunit (Qin *et al*. 1998). The α_2_δ subunits also increased the rate of inactivation to varying extents, with the greatest effect being observed for α_2_δ‐1 (Fig. 3 *C*; for review see Canti *et al*. 2003).

Thus, although the α_2_δ subunits affect the kinetics and voltage‐dependent properties of the different calcium channel isoforms, there is no clear consensus for the different α_1_ and α_2_δ isoform combinations. One origin of this complexity may be that there are also usually more mature channels in the plasma membrane in the presence of α_2_δ subunits. Such a diversity of effects, although they may appear subtle when measured in isolation, can have important consequences in terms of calcium‐and voltage‐dependent events in cells, including action potential shape (Hoppa *et al*. 2012, 2014), and the firing properties of neurons (Margas *et al*. 2016).

## Splice variants of α_2_δ subunits

The main α_2_δ‐1 subunit splice variant present in rat brain is different from that seen in skeletal muscle (Kim *et al*. 1992). Sequence alignments identified alternative splicing in three regions, called A, B and C (Angelotti & Hofmann, 1996). Our recent study (Lana *et al*. 2014) indicates that regions A and B are in separate exons, with region A in rat being encoded by exon 18a and region B representing an alternative 3′ splice acceptor site (start site) of exon 19. Region C is also a cassette exon. The main splice variant in rat skeletal muscle is +A +B ΔC, whereas α_2_δ‐1 (ΔA + B + C) is the principal brain splice variant (Angelotti & Hofmann, 1996; Lana *et al*. 2014). We have recently shown that it is also the main splice variant in DRG neurons (Lana *et al*. 2014). However, we also identified a novel minor splice variant (α_2_δ‐1 ΔA + B ΔC) in these neurons (Lana *et al*. 2014). Alternative splicing of other α_2_δ subunits has been described in other studies (Barclay & Rees, 2000; Qin *et al*. 2002).

## Distribution of α_2_δ subunits in the peripheral and central nervous systems

The α_2_δ‐1, α_2_δ‐2 and α_2_δ‐3 subunits are widely expressed in both the peripheral and central nervous system, as documented in a comprehensive *in situ* hybridization study (Cole *et al*. 2005). α_2_δ‐1 is present in many neuronal cell types (Cole *et al*. 2005), including DRG neurons (Newton *et al*. 2001; Bauer *et al*. 2009). The α_2_δ‐1 protein is mainly situated in presynaptic terminals, as well as, to smaller extent, in neuronal somata, and also in dendrites (Taylor & Garrido, 2008; Bauer *et al*. 2009).

The α_2_δ‐1 transcript is expressed preferentially in excitatory compared to inhibitory neurons (Cole *et al*. 2005). In contrast, α_2_δ‐2 expression was found to be lower than α_2_δ‐1 in most brain regions, with restricted areas showing significant expression, such as the cerebellum (Cole *et al*. 2005). The distribution of α_2_δ‐2 partially correlates with expression in GABAergic neurons, including cerebellar Purkinje neurons (Barclay *et al*. 2001; Cole *et al*. 2005). The α_2_δ‐3 transcript is present throughout the brain, and is particularly prevalent in the caudate‐putamen (Cole *et al*. 2005). It is also present in the auditory system (Pirone *et al*. 2014) and in the retina (Perez de Sevilla *et al*. 2015). In contrast, α_2_δ‐4 protein is found in certain endocrine tissues, and is expressed at a low level in the brain (Qin *et al*. 2002). It also plays a key role in the retina (De Sevilla Muller *et al*. 2013).

## Role of α_2_δ‐1 in neuropathic pain

Neuropathic pain is chronic pain resulting from nerve damage, which may have a number of different underlying causes. Neuropathic pain can be a result of trauma, either directly damaging or impinging on axons. Trigeminal neuralgia, which involves severe facial and jaw pain, is often caused by trapping or pressure on sensory nerves. Cancer‐induced neuropathic pain can be also result from direct damage to sensory nerves, or activation of nociceptors as a result of mediators secreted from tumours or in the inflammatory response (Schmidt *et al*. 2010). Neuropathic pain can also commonly be caused by direct damage to nerves by toxins and drugs. This would include diabetic neuropathy, due to axon damage as a direct result of chronic elevated plasma glucose concentration, and neuropathy caused by cancer chemotherapeutic drugs, for example platinum‐based drugs such as cisplatin, microtubule‐disrupting taxanes, such as paclitaxel, and vinca alkaloids including vincristine. Some older anti‐human immunodeficiency virus (HIV) drugs, such as 2′,3′‐dideoxycytidine, can also result in nerve damage and neuropathic pain (Joseph *et al*. 2004). Viral infection of DRGs can also cause neuralgia, including chronic post‐herpetic neuralgia (following shingles), or HIV‐induced neuropathic pain, which can be mimicked by injection of the viral coat protein HIV gp‐120 (Wallace *et al*. 2007; Schutz & Robinson‐Papp, 2013). Thus both HIV infection and some of the treatments used may initiate neuropathic damage.

Sensory nerve injury results in a change in transcription in those damaged neurons of many genes, which may be either up‐ or down‐regulated, often many‐fold (Newton *et al*. 2001; Wang *et al*. 2002; Xiao *et al*. 2002; Dawes *et al*. 2014). The mechanism of this effect has been investigated for the chemotherapeutic agent paclitaxel and may involve injury‐induced modulation of Ca^2+^ entry and neuronal calcium sensor‐1 degradation (Boehmerle *et al*. 2006, 2007).

Among the large number of genes whose expression is altered, there is a consistent elevation of α_2_δ‐1 mRNA, shown by *in situ* hybridization (Newton *et al*. 2001), quantitative PCR (Bauer *et al*. 2009), microarray analysis (Wang *et al*. 2002; Xiao *et al*. 2002) and RNAseq (Perkins *et al*. 2014). There is an equivalent increase in α_2_δ‐1 protein in DRGs and in the dorsal horn of the spinal cord, shown by immunoblotting (Luo *et al*. 2001) and immunohistochemistry (Bauer *et al*. 2009). The increase in α_2_δ‐1 appears to occur in every damaged DRG neuron (Bauer *et al*. 2009; Patel *et al*. 2013). In contrast, the levels of Ca_V_2.2 mRNA and protein are not altered in these models (Wang *et al*. 2002; Li *et al*. 2006), although a change in splicing of exon 37 has been documented (Altier *et al*. 2007). This leads to the hypothesis that elevated α_2_δ‐1 results in increased Ca_V_2.2 trafficking to terminals or localization to active zones, thus affecting presynaptic function. Nevertheless, α_2_δ‐1 may also have other roles, for example in neuronal sprouting.

Transgenic mice that overexpress α_2_δ‐1 exhibit a baseline phenotype of allodynia and hyperalgesia (Li *et al*. 2006), suggesting that the α_2_δ‐1 level in DRG neurons is important for determining the neuropathic response. In agreement with these results, we have shown that in α_2_δ‐1 knockout mice (Fuller‐Bicer *et al*. 2009), there is a marked reduction in baseline responses to mechanical and cold stimulation, and a very retarded hyperalgesic response to sciatic nerve injury, in comparison to wild‐type littermate mice (Patel *et al*. 2013). In agreement with this we found that DRGs from α_2_δ‐1 knockout mice showed strongly reduced ability to fire multiple action potentials (Margas *et al*. 2016).

**Figure 5:**
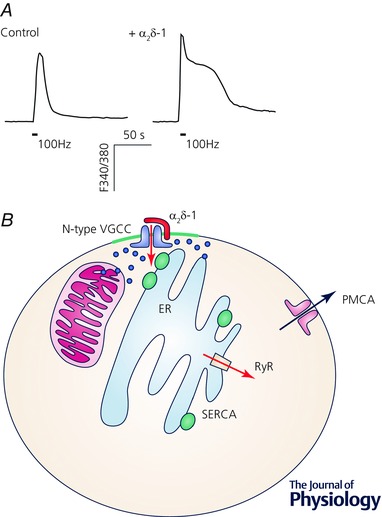
**Effect of α_2_δ‐1 on cytosolic Ca^2+^ levels** *A*, overexpression of α_2_δ‐1 in DRG neurons increased the width of depolarization‐induced intracellular calcium transients, measured using Fura‐2, induced by 100 Hz electrical stimulation (indicated by the bar). Data are taken from a recent study (D'Arco *et al*. 2015). *B*, cartoon of localization of Ca_V_2.2 (N‐type) calcium channels in the plasma membrane near to ER and mitochondria. Ca^2+^ is taken up into ER via the sarcoplasmic–endoplasmic reticulum Ca^2+^‐ATPase (SERCA) pump, and can be released by ryanodine receptor (RyR) activation. Ca^2+^ is pumped out of cells by the plasma membrane Ca^2+^‐ATPase (PMCA). Cartoon adapted from from a recent study (D'Arco *et al*. 2015).

We have also recently shown that heterologous over‐expression of α_2_δ‐1 in cultured DRG neurons (to mimic *in vitro* the neuropathic state) leads to increased calcium currents and prolonged cytoplasmic Ca^2+^ responses resulting from membrane depolarization (Fig. 5 *A*). These prolonged Ca^2+^ transients, once initiated, are not dependent on extracellular Ca^2+^ but are buffered by mitochondria. Thus, by controlling Ca_V_2.2 channel density in the plasma membrane, possibly at sites where mitochondria and ER are also closely apposed, the α_2_δ‐1 subunit has a large effect on depolarization‐induced intracellular Ca^2+^ signalling in DRG neurons (D'Arco *et al*. 2015; Fig. 5 *B*).

Regarding the involvement of other α_2_δ subunits in the pain pathway, the *Drosophila melanogaster CACNA2D3* homologue, *straitjacket*, was identified as a gene involved in pain processing (Neely *et al*. 2010).

## Role of α_2_δ subunits in epilepsies

Prior to the identification of α_2_δ‐1 as the receptor for gabapentin (see below), this drug was known to be of use in the treatment of some forms of epilepsy, as an adjunct drug to improve seizure control (Marson *et al*. 2000). Gabapentin binds to both α_2_δ‐1 and α_2_δ‐2, but not to the other α_2_δ subunits. Subsequently, we found, together with Michele Rees and Mark Gardiner, that the mutant mouse strains *ducky* and *ducky^2J^* involved disruption of the *cacna2d2* gene (Barclay *et al*. 2001). These mice display paroxysmal dyskinesia and absence seizures. Although the mutations are different in the two mouse strains, being a complex rearrangement of the gene in *ducky* and a two base pair deletion in *ducky^2J^*, no full length α_2_δ‐2 protein is produced in either strain (Barclay *et al*. 2001; Brodbeck *et al*. 2002; Donato *et al*. 2006). Another mutant mouse, *entla*, with a similar epileptic phenotype, was then identified and found to have a duplication of exon 3 in *cacna2d2* (Brill *et al*. 2004). Mice with a targeted gene deletion in *cacna2d2* also show an epileptic and ataxic phenotype (Ivanov *et al*. 2004). The mutation in *ducky* and *ducky^2J^* mice is associated with abnormal morphology of the Purkinje cells (Brodbeck *et al*. 2002) and markedly attenuated spontaneous activity in these neurons (Donato *et al*. 2006).

Two human family pedigrees have recently been investigated, in which homozygous recessive mutations in *CACNA2D2* resulted in infantile epileptic encephalopathy (Edvardson *et al*. 2013; Pippucci *et al*. 2013). The carriers of a single copy of the mutations had no phenotype, in agreement with the absence of phenotype in mice heterozygous for *cacna2d2* expression (Barclay *et al*. 2001).

For α_2_δ‐1, no central phenotypes have been identified with any certainty in humans, possibly because most neurons contain more than one subtype of α_2_δ subunit, and these proteins may have a partially interchangeable function. However, *CACNA2D1* has been identified as a candidate gene associated with some cases of West syndrome, an early‐onset epileptic encephalopathy (Hino‐Fukuyo *et al*. 2015). The *CACNA2D1* locus has also been implicated in three patients investigated with intellectual disability and epilepsy, although these patients had deletions that also affected other genes (Vergult *et al*. 2015).

## Night blindness

Mutations in the gene *CACNA2D4*, encoding α_2_δ‐4, produce photoreceptor dysfunction, resulting in a form of night blindness (Wycisk *et al*. 2006 *b*). A spontaneously occurring mouse mutation has also been identified in this gene, with a phenotype of autosomal recessive cone dystrophy, again causing night blindness (Wycisk *et al*. 2006 *a*,*b*). This emphasizes the importance of α_2_δ‐4 in photoreceptor function.

## Neuropsychiatric disorders

As we have reviewed recently (Heyes *et al*. 2015), rare deleterious mutations in many of the calcium channel genes including *CACNA2D1, CACNA2D2* and *CACNA2D4* have been linked to both bipolar disorder and schizophrenia (Purcell *et al*. 2014). Furthermore, *CACNA2D2* and *CACNA2D4* have also been linked to these psychiatric disorders in Genome‐Wide Association Studies (Cross‐Disorder Group of the Psychiatric Genomics Consortium, 2013). However, most of the single nucleotide polymorphisms (SNPs) that are associated with these disorders are in introns or intergenic regions, and it remains unclear whether the SNPs have any effects to increase or decrease overall expression, or expression of particular splice variants, or otherwise alter the function of the gene with which they are associated (Heyes *et al*. 2015). Nevertheless, it has recently been found that expression of *CACNA1S, CACNA2D4* and *CACNA1F* were increased in hippocampal‐like neurons derived from induced pluripotent stem cells in patients with bipolar disorder (Mertens *et al*. 2015). It is interesting that these particular calcium channel genes normally show very low expression in brain, so their physiological role in hippocampus is unclear.

A *CACNA2D3* splice site mutation was identified as one of a large number of ‘likely gene‐disrupting mutations’ involved in autism specrum disorders (Iossifov *et al*. 2012). Other rare germline mutations, introducing premature stop codons or aberrant splicing, predicting truncated proteins, have also been found to be associated with autism (Girirajan *et al*. 2013; De Rubeis *et al*. 2014). Given the likelihood that autism involves synaptic dysfunction (Malhotra & Sebat, 2012; Ting *et al*. 2012), it is perhaps not surprising that mutations in α_2_δ‐3, which is present in presynaptic terminals, are found to be one of many potential genetic causes of autism.

## Cardiac and endocrine dysfunction

The α_2_δ‐1 protein is strongly expressed together with the L‐type calcium channels in skeletal, cardiac and smooth muscle (Ellis *et al*. 1988; Jay *et al*. 1991; Klugbauer *et al*. 1999; Wolf *et al*. 2003; Walsh *et al*. 2009). *CACNA2D1* mutations have been identified to cause human cardiac dysfunction, including short QT syndrome (Templin *et al*. 2011) and Brugada syndrome (Burashnikov *et al*. 2010). The mechanism of disruption resulting from mutations in α_2_δ‐1 has been probed (Bourdin *et al*. 2015). In agreement with this, disruption of the *cacna2d1* gene in mice also caused a cardiac phenotype; the mice exhibited a reduction in basal ventricular myocardial contractility, associated with lower cardiac calcium current density (Fuller‐Bicer *et al*. 2009). Furthermore, mice lacking α_2_δ‐1 also showed reduced pancreatic β‐cell calcium currents, and an increased tendency to develop diabetes, particularly on one genetic background (Tuluc *et al*. 2014).

## α_2_δ subunits as a therapeutic target

The α_2_δ subunits were discovered to be therapeutic targets completely fortuitously, by virtue of being the unexpected protein target for gabapentin binding. Otherwise they would not have been considered *a priori* as a relevant drug target, because of the absence of any known ligand or mechanism of action.

## Identification of α_2_δ subunits as gabapentin receptors

Gabapentin and pregabalin were first synthesized as analogues of GABA, with the aim of developing novel antiepileptic drugs (Taylor *et al*. 2007; Silverman, 2008). After it was found that they did not act via GABA pathways, purification of the brain ^3^H‐gabapentin ‘receptor’ then resulted in the surprise identification of α_2_δ‐1 (Gee *et al*. 1996; Brown *et al*. 1998; Brown & Gee, 1998; Field *et al*. 2006; Li *et al*. 2011). ^3^H‐Gabapentin also binds to α_2_δ‐2 (Gong *et al*. 2001). Several residues in α_2_δ‐1 and α_2_δ‐2 are involved in the binding of the gabapentinoid drugs; one important motif involves three arginine residues, just proximal to the VWA domain (Brown & Gee, 1998; Davies *et al*. 2006; Field *et al*. 2006). The binding pocket for gabapentin in α_2_δ‐1 has been further elucidated in the cryo‐EM structure (Wu *et al*. 2015). One may speculate that the basis of the binding of these drugs to α_2_δ‐1 and α_2_δ‐2 subunits might stem from the presence of the Cache domains, and their ancestral role to sense nutrients in bacteria. Furthermore, it is likely that a low molecular weight endogenous ligand might also bind to α_2_δ‐1 and α_2_δ‐2, and be displaced competitively by gabapentin. The binding affinity for ^3^H‐gabapentin increases progressively as the α_2_δ protein is purified or dialysed, or when isolated in lipid raft fractions, suggesting that an endogenous bound substance that competes with gabapentin binding is being removed (Brown *et al*. 1998; Davies *et al*. 2006; Lana *et al*. 2016). It is also possible that gabapentin binding might disrupt the function(s) of the VWA domain or the Cache domains (Dolphin, 2012; Cassidy *et al*. 2014). It would be of great interest to determine the nature and function of this endogenous small molecule.

## Use of gabapentinoid drugs for epilepsy

Gabapentin is licensed for use as an adjunct drug in several types of epilepsy (Marson *et al*. 2000) and as a monotherapy in some partial‐onset seizures (Glauser *et al*. 2006). Pregabalin is also effective in the therapy of some epilepsies (for review see Taylor *et al*. 2007). In order to determine whether α_2_δ‐1 or α_2_δ‐2 was responsible for the anti‐epileptic effects of these drugs, experiments were performed using knock‐in mice, engineered to contain a mutant α_2_δ‐1 or α_2_δ‐2 with reduced affinity for gabapentinoid drug binding (Field *et al*. 2006; Lotarski *et al*. 2011). Pregabalin was not found to be effective against electroshock‐induced seizures in mice in which α_2_δ‐1 subunits are mutated, whereas it was still effective in mice with an equivalent mutation in α_2_δ‐2 (Lotarski *et al*. 2014); thus it is likely that the anti‐seizure effect of these drugs is primarily via binding to α_2_δ‐1.

## Neuropathic pain and the role of α_2_δ subunits

Gabapentin and pregabalin are licensed for use in the treatment of various forms of neuropathic pain (Taylor *et al*. 2007). In contrast, they have no effect on acute nociceptive pain (Dickenson *et al*. 2005; Moore *et al*. 2009). In neuropathic pain models in rodents, it has been shown that binding of the gabapentinoid drugs to α_2_δ‐1 subunits is required for their therapeutic effect (Field *et al*. 2006). This finding indicates that binding to α_2_δ‐2 is not important for the effect of gabapentin, and, indeed, α_2_δ‐2 was found to be reduced rather than up‐regulated in injured DRG neurons (Bauer *et al*. 2009). Pregabalin is also used in the treatment of fibromyalgia, defined as generalized widespread pain, which may also have a neuropsychiatric aspect (Smith & Moore, 2012).

In a recent study, we have documented changes in splicing, in addition to overall up‐regulation of α_2_δ‐1, in injured rat DRG neurons (Lana *et al*. 2014). There was elevated expression of a novel splice variant (α_2_δ‐1 ΔA + B ΔC), which has a lower affinity for gabapentin (Lana *et al*. 2014). It is interesting to speculate that variable up‐regulation of this, or other, splice variants in people who develop neuropathic pain might be relevant to the inconsistent efficacy of the α_2_δ ligand drugs within the patient population.

## Calcium channel currents: effects of gabapentinoid drugs

Small acute inhibitory effects of gabapentin have been observed on calcium currents in several systems (Stefani *et al*. 1998; Martin *et al*. 2002; Sutton *et al*. 2002). However, in other studies no acute responses to gabapentin have been reported on native or heterologously expressed calcium channel currents (Schumacher *et al*. 1998; Davies *et al*. 2006; Heblich *et al*. 2008; Hendrich *et al*. 2008). In DRGs from α_2_δ‐1‐overexpressing mice, it was observed that the calcium currents were rapidly inhibited by gabapentin, whereas this was not the case in wild‐type mice (Li *et al*. 2006). These results imply either that gabapentin is not a direct channel blocker, which would indeed be predicted from the location of its binding site, or that α_2_δ‐1 is not associated with all the relevant calcium channels in DRGs from wild‐type mice.

## Calcium channel trafficking: effects of gabapentinoid drugs

**Figure 6:**
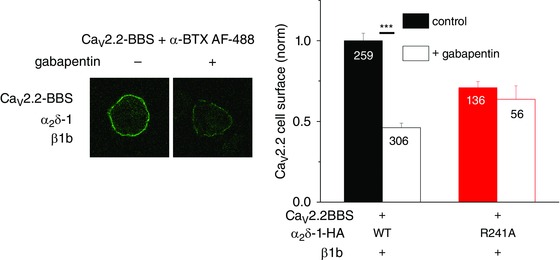
**Ca_V_2.2 cell surface expression: effect of gabapentin** Cell surface expression of bungarotoxin binding site (BBS) tagged Ca_V_2.2 labelled with α‐bungarotoxin (BTX) coupled to AF488 dye (green). Left panel: examples of N2a cells transfected with Ca_V_2.2/β1b/α_2_δ‐1 in the absence (left), and presence (right) of gabapentin (100 μm for 24 h). Right panel: mean (± SEM) data for cell surface expression of Ca_V_2.2, for cells expressing Ca_V_2.2/β1b/α_2_δ‐1 (black‐filled and open bars), Ca_V_2.2/β1b/α_2_δ‐1 R241A (a mutant α_2_δ‐1 that does not bind gabapentin; red‐filled and open bars) in the absence (filled bars), and presence (open bars) of gabapentin 100 μm for 24 h. Data are taken from a recent study (Cassidy *et al*. 2014). The number of cells measured is indicated on the bars, ^***^ *P*<0.001, Student's *t* test.

We have found that incubation of cultured cells for several hours or days, rather than acute application of gabapentin, produces a reduction of calcium currents, both in expression systems when α_2_δ‐1 or α_2_δ‐2 was co‐expressed, and also in DRG neurons (Heblich *et al*. 2008; Hendrich *et al*. 2008; Tran‐Van‐Minh & Dolphin, 2010). We observed a corresponding reduction in expression of the α_2_δ and associated α_1_ subunits on the cell surface (Hendrich *et al*. 2008; Tran‐Van‐Minh & Dolphin, 2010; Cassidy *et al*. 2014; Fig. 6). We also found that gabapentin reduced forward trafficking of α_2_δ‐2 by inhibiting a post‐Golgi trafficking step, in a process requiring Rab11, which is involved in trafficking of cargoes in the recycling endosome compartment (Tran‐Van‐Minh & Dolphin, 2010). When this pathway was isolated, a response to gabapentin could be observed on a time‐scale of 30 min. Furthermore, we observed that chronic administration to nerve‐injured rats of an anti‐hyperalgesic dosing regimen of pregabalin reduced the elevation in the dorsal horn of presynaptic α_2_δ‐1. We interpreted this effect as being due to reduced axonal trafficking *in vivo* (Bauer *et al*. 2009). It is possible that gabapentinoid drugs selectively target calcium channel populations that are rapidly turning over, thus sparing skeletal muscle and cardiac channels, but this will need further experimentation.

## Binding of α_2_δ subunits to other proteins: effects of gabapentinoid drugs

In various tissues it has been found that a proportion of α_2_δ subunits can be purified by biochemical means separately from α_1_ subunits (Gee *et al*. 1996; Müller *et al*. 2010), indicating that they may be only loosely associated with α_1_ subunits, or may exist separately. This suggests that they may have other functions in addition to being calcium channel subunits. For example, the α_2_δ‐3 proteins have a documented role in formation of synaptic boutons in *Drosophila*, which was found to be independent of their involvement with calcium channels, in that it was not mimicked by deletion of the relevant α_1_ subunit (Kurshan *et al*. 2009). However, since α_2_δ subunits play a role in trafficking calcium channels, as well as in calcium channel function, it may be that α_2_δ subunits directly influence the calcium transients which are involved in neurite outgrowth and synapse formation during development (Gu *et al*. 1994).

Furthermore, the α_2_δ‐1 protein has been found to co‐immunoprecipitate with thrombospondins, which are large multi‐domain extracellular matrix proteins (Eroglu *et al*. 2009); although it should be noted that thrombospondins also bind to many other proteins (Kazerounian *et al*. 2008). In the brain, specific thrombospondins are produced by astrocytes and promote the formation of silent excitatory synapses, lacking postsynaptic receptors (Christopherson *et al*. 2005). Thrombospondin‐induced synaptogenesis was found to require the postsynaptic presence of α_2_δ‐1 (Eroglu *et al*. 2009). Gabapentin was found to disrupt the *in vitro* interaction between α_2_δ‐1 and the synaptogenic domain of thrombospondin‐2, and also disrupted synaptogenesis, although it had no effect on pre‐formed synapses (Eroglu *et al*. 2009). This effect on synaptogenesis may not be relevant to the main mechanism of action of gabapentin either in neuropathic pain or as an antiepileptic drug, as much synaptic sprouting and remodelling would have taken place before the onset of therapy, although gabapentin could have a protective effect via this mechanism. Nevertheless, it should be emphasized that birth defects were found to be extremely uncommon in babies following chronic gabapentin exposure in the uterus of mothers who were taking the drug as an anti‐epileptic medication (Morrow *et al*. 2006; Molgaard‐Nielsen & Hviid, 2011), suggesting that it does not have any significant effect on synapse formation during development *in utero*.

As a corollary of a potential interaction between α_2_δ‐1 and thrombospondins, we have recently examined whether interaction of thrombospondins with α_2_δ‐1 might influence ^3^H‐gabapentin binding (Lana *et al*. 2016). We used thrombospondin‐4 as it is upregulated in neuropathic pain models (Pan *et al*. 2015). We found that in membranes from co‐transfected cells, thrombospondin‐4, significantly reduced the affinity for ^3^H‐gabapentin binding to α_2_δ‐1, in a divalent cation‐dependent manner. However, the effect on ^3^H‐gabapentin binding was not reproduced by the synaptogenic domain of thrombospondin‐4. Furthermore, we found only weak co‐immunoprecipitation of the two proteins, which could not be reproduced with the synaptogenic domain of thrombospondin‐4 (Lana *et al*. 2016). We also could not demonstrate any association between α_2_δ‐1 and thrombospondin‐4 on the cell surface of transfected cells, suggesting that the interaction between these two proteins to disrupt ^3^H‐gabapentin binding is occurring in an intracellular compartment of the transfected cells (Lana *et al*. 2016). It is nevertheless possible that such an interaction might reduce the efficacy of gabapentin in patients.

## Conclusions and future directions

The α_2_δ subunits are important auxiliary subunits of the Ca_V_1 and Ca_V_1 voltage‐gated calcium channels. They play major roles in trafficking of these channels, both to the plasma membrane and to specific domains, as well as influencing the activation and biophysical properties of these channels. The mechanism of these effects, at a cell biological level, still remains to be determined in detail. They also play a role in the pathology of a number of genetic and other diseases, and represent an important therapeutic target site for drugs. Future therapeutic directions are likely to include identifying selective antagonists distinguishing Ca_V_1.3 from Ca_V_1.2 and other L‐type channels, finding selective antagonists for the different T‐type channels, and understanding better the mechanism of action of the α_2_δ ligands.

## Additional information

### Competing interests

None declared.
